# Metabolic Reprogramming and Epithelial-Mesenchymal Plasticity: Opportunities and Challenges for Cancer Therapy

**DOI:** 10.3389/fonc.2020.00792

**Published:** 2020-05-20

**Authors:** Nai-Yun Sun, Muh-Hwa Yang

**Affiliations:** ^1^Institute of Clinical Medicine, National Yang-Ming University, Taipei, Taiwan; ^2^Cancer Progression Research Center, National Yang-Ming University, Taipei, Taiwan; ^3^Division of Medical Oncology, Department of Oncology, Taipei Veterans General Hospital, Taipei, Taiwan

**Keywords:** cancer metabolism, aerobic glycolysis, epithelial-mesenchymal plasticity, metastasis, drug resistance

## Abstract

Metabolic reprogramming and epithelial-mesenchymal plasticity are both hallmarks of the adaptation of cancer cells for tumor growth and progression. For metabolic changes, cancer cells alter metabolism by utilizing glucose, lipids, and amino acids to meet the requirement of rapid proliferation and to endure stressful environments. Dynamic changes between the epithelial and mesenchymal phenotypes through epithelial-mesenchymal transition (EMT) and mesenchymal-epithelial transition (MET) are critical steps for cancer invasion and metastatic colonization. Compared to the extensively studied metabolic reprogramming in tumorigenesis, the metabolic changes in metastasis are relatively unclear. Here, we review metabolic reprogramming, epithelial-mesenchymal plasticity, and their mutual influences on tumor cells. We also review the developing treatments for targeting cancer metabolism and the impact of metabolic targeting on EMT. In summary, understanding the metabolic adaption and phenotypic plasticity will be mandatory for developing new strategies to target metastatic and refractory cancers that are intractable to current treatments.

## Background: Adaptions of Cancer Cells for Tumor Growth and Metastasis

Cancer cells are characterized by rapid proliferation and metastasis (1). Adaptation of cancer cells to stressful environments is mandatory to ensure their growth and metastasis. Cancer cells utilize metabolic reprogramming to meet their energy requirements for growth (2), whereas the dynamic changes between epithelial and mesenchymal states are important for the successful development of metastatic tumors (3). Understanding the relationship between these two events is not only scientifically interesting but also important for developing strategies to target metastatic cancers.

Metabolic rewiring allows uncontrolled proliferative cancer cells to meet their requirements for energy-demanding activities and macromolecule biosynthesis (1, 2). Cancer cells are dependent on exogenous nutrients because endogenous nutrients are insufficient to maintain their active proliferation (4–6). Blockage of cancer-specific metabolism retards tumor growth or induces cancer cell death through modulation of various signaling pathways (7–10). Compared to the extensive understanding of metabolic reprogramming during the carcinogenic process, knowledge about the metabolic changes of cancer cells in late-stage progression and metastasis is relatively limited.

Epithelial-mesenchymal transition (EMT) is a process in which epithelial cells lose their intercellular adherence and cellular polarity and acquire the mesenchymal phenotype (11). EMT is a crucial mechanism for embryogenesis, organ fibrosis, and cancer metastasis (11). In cancer cells, activation of EMT leads cells to acquire migration, invasion, stemness, and drug resistance, whereas the reverse process of EMT, i.e., mesenchymal-epithelial transition (MET), is important for metastatic colonization (3). The updated concept suggests that dynamic changes between epithelial and mesenchymal phenotypes, i.e., epithelial-mesenchymal plasticity, rather than a fixed phenotype, are preferred for developing metastatic tumors, and a hybrid epithelial-mesenchymal state harbors a higher plasticity for metastasis (12).

Here, we will review the metabolic changes and current strategies for targeting cancer metabolism, epithelial-mesenchymal plasticity, and the mutual influences between these two events. The impact of metabolic targeting on epithelial/mesenchymal phenotypes and metastasis will also be reviewed.

## Metabolic Reprogramming in Cancer Cells: Mechanisms, Biomarkers, and Therapeutic Targeting

### Mechanism for Metabolic Changes in Cancer Cells

Cells break down nutrients to generate energy and building blocks through metabolic pathways. Adenosine triphosphate (ATP), as the cellular energy currency, is generated by aerobic or anaerobic respiration. Cells take up glucose and convert it into pyruvate through glycolysis. Under normoxic environments, cells further convert pyruvate into acetyl coenzyme A (acetyl-CoA) in mitochondria, which provides an acetyl group to the tricarboxylic acid (TCA) cycle, a major reaction for energy generation. Under hypoxic conditions, cells convert pyruvate into lactate through anaerobic glycolysis. However, cancer cells produce lactate regardless of oxygen availability (13), and this phenomenon is called the Warburg effect or aerobic glycolysis (14, 15). Cancer cells generate ATP and glycolytic intermediates quickly through only 10 reaction steps within aerobic glycolysis compared to the highly complicated oxidative phosphorylation. ^18^F-deoxyglucose-positron emission tomography confirmed the increased glucose uptake in tumors compared to normal tissues in cancer patients (16). Interestingly, cancer cells do not completely depend on aerobic glycolysis; instead, they also maintain functioning mitochondria and oxidative phosphorylation (17, 18). A possible explanation is that the mitochondrial electron transport chain generates high levels of reactive oxygen species (ROS) during oxidative phosphorylation of cancer cells. ROS activate signaling pathways to stimulate cancer cell proliferation (18). Cancer cells also generate high levels of nicotinamide adenine dinucleotide phosphate (NADPH) as an antioxidant in both the mitochondria and the cytosol to limit excessive ROS to prevent ROS-induced apoptosis (19). Both the Warburg effect and oxidative phosphorylation generate sufficient energy and glycolytic carbon intermediates, which are essential for the synthesis of macromolecules to meet the requirements of highly proliferative cancer cells. In addition to the derangement of glucose metabolism, mutations of the key enzymes in TCA cycle/glucose metabolism have been identified in human cancers. Isocitrate dehydrogenase (IDH) includes three main isoforms. IDH3 is the main isoform in the TCA cycle that catalyzes the irreversible conversion of isocitrate to α-ketoglutarate in the mitochondria and generates NADH. IDH1 and IDH2 generate NADPH by catalyzing the reversible isocitrate-to-α-ketoglutarate conversion in the cytoplasm and the mitochondria, respectively. Mutations of *IDH1* and *IDH2* have been recognized as oncogenic events through decreasing α-ketoglutarate and increasing D-2-hydroxyglutarate production (20), and the neomorphic *IDH* mutant has been shown in acute myeloid leukemia (21) and gliomas (22).

In addition to aerobic glycolysis, there are several major metabolic derangements noted in cancer cells. The pentose phosphate pathway (PPP) is recognized as an important pathway for catabolizing glucose in cancer cells. The PPP is important because it not only utilizes glucose for energy but also maintains the biosynthesis of lipids and nucleotides and the antioxidant responses of cancer cells (23). Furthermore, reprogramming of lipid metabolism is an important feature of cancer cells. Oxidation and synthesis of lipids support cancer cell proliferation by providing building blocks for membrane synthesis and additional energy sources (24). Fatty acids are mostly obtained from environmental sources in normal cells; in contrast, *de novo* synthesis of fatty acids is frequently increased in cancer cells (25). Another well-recognized metabolic alteration in cancer cells is glutamine dependency. Glutamine not only provides an important metabolite in the TCA cycle (α-ketoglutarate by glutaminase) (26) but also provides the nitrogen building blocks for nucleotide and amino acid synthesis (2).

Deregulation of nucleotide metabolism, especially ATP, has also been noted as a major event in cancer metabolism, and it mainly influences antitumor immunity. High levels of extracellular ATP generation are induced by inflammation, ischemia, or hypoxia within tumor microenvironments through various pathways, including channel or transporter-mediated release, vesicular exocytosis, or direct release due to cell destruction (27). Extracellular ATP is sequentially converted to adenosine monophosphate (AMP), and AMP is hydrolyzed to adenosine through ectonucleotidase CD39- and CD73-mediated dephosphorylation (28). Adenosine is not only involved in cancer growth but also generates anti-inflammatory responses by modulating various cells in the tumor microenvironment, such as endothelial cells, mast cells, natural killer cells, neutrophils, macrophages, dendritic cells, and lymphocytes (29). In addition, adenosine stimulates the differentiation of naive CD4^+^CD25^−^ T cells to CD4^+^CD25^+^Foxp3^+^ regulatory T cells and induces T-cell anergy (30). Notably, HIF-1α induced by the hypoxic tumor microenvironment enhances the expression of adenosinergic molecules, including CD39 and CD73, as well as the adenosine 2B receptor (A2BR) (31, 32). Overexpression of these adenosinergic molecules is associated with metastasis and poor patient outcomes in different cancers (28, 33).

Thus, the metabolic reprogramming of cancer cells includes aerobic glycolysis, the PPP, lipid metabolism changes, glutaminolysis, nucleotide metabolism, and many other events. These adaptive changes provide sufficient energy for sustaining cancer cell proliferation, providing building blocks for macromolecule synthesis, and suppressing antitumor immunity for immune evasion.

### Therapeutic Targeting for Cancer Metabolism

Canonical cancer treatments preferentially target proliferation-related pathways with unavoidable toxicity to proliferating normal cells such as intestinal crypt cells, hematopoietic cells, and hair follicle cells. In addition, certain normal cells exhibit a higher proliferation rate than cancer cells (34). Targeting tumor-specific metabolism is therefore an attractive strategy for anticancer treatment. However, the complex crosstalk between tumor cells and the microenvironments substantially increases the difficulty of specific targeting of cancer metabolism. For example, lactate produced by cancer cells shuttles not only to neighboring cancer cells but also to the surrounding stromal cells and vascular endothelial cells (35). Here, we review the recent progress in targeting cancer metabolism, including the amino acid catabolism and the metabolism of lipids and glucose. Preclinical and clinical studies targeting cancer metabolism are summarized in Table 1.

**Table 1:** Developing treatments for targeting cancer metabolism.

**Drug**	**Target**	**Pathway**	**Cancer type**	**Stage**	**References**
CB-839	GLS1	Gln	TNBC	Pre-clinical	(36)
CB-839	GLS1		LC	Pre-clinical	(37)
CB-839	GLS1		CRC	Phase II	(38)
CB-839	GLS1		Solid tumors	Phase I	(39)
CB-839	GLS1		RCC	Phase II	(40)
GPNA	ASCT2		NSCLC	Pre-clinical	(41)
Ab3-8 mAb	ASCT2		CRC	Pre-clinical	(42)
L-ASNase	Asn	Asn	BC	Pre-clinical	(43)
L-ASNase	Asn		ALL	FDA approval	(44, 45)
PEG-BCT-100	Arg	Arg	HCC	Phase I	(46)
ADI-PEG 20	Arg		HCC	Phase III	(47)
SCH58261	A2AR	Ado	Solid tumors	Pre-clinical	(48)
MEDI9447	CD73		Solid tumors	Phase I	(49)
CPI-444	A2AR		Solid tumors	Phase I	(50)
CPI-006	CD73		Solid tumors	Phase I	(51)
TVB-2640	FASN	Lipid synthesis	BC	Phase II	(52)
TVB-2640	FASN		MA	Phase II	(53)
ND-646	ACC		NSCLC	Pre-clinical	(54)
ND-654	ACC	Lipid synthesis	HCC	Pre-clinical	(55)
AZD3965	MCT	Glycolysis	Solid tumors	Phase I	(56)
Enasidenib	IDH2	TCA cycle	AML	FDA approval	(57)
Ivosidenib	IDH1		AML	FDA approval	(58)
CPI-613	PDH		NSCLC, PaC	Pre-clinical	(59)
CPI-613	PDH		PaC	Phase I	(60)

*TNBC, triple-negative breast cancer; LC, lung cancer; CRC, colorectal cancer; RCC, renal cell carcinoma; NSCLC, non-small cell lung cancer; BC, breast cancer; ALL, acute lymphoblastic leukemia; HCC, hepatocellular carcinoma; MA, malignant astrocytoma; AML, acute myeloid leukemia; PaC, pancreatic cancer; Gln, glutamine; Asn, asparagine; Arg, arginine; Ado, adenosine*.

## Amino Acid Metabolism

### Glutamine

Glutamine addiction has been extensively found in cancer cells (61). Glutamine is involved in various metabolic processes of cancer cells: glutamine acts as a nitrogen donor for nucleic acid and amino acid biosynthesis, drives oxidative phosphorylation and is the substrate for lipid and glutathione synthesis (62). Moreover, Muir et al. showed that glutamine is probably a more important substrate *in vitro* in cell culture than *in vivo* (63). There are two strategies for targeting glutamine metabolism in cancer cells: inhibition of glutaminase that can convert glutamine into glutamate and blockage of the major glutamine transporter alanine-serine-cysteine transporter 2 (ASCT2) to suppress the influx of glutamine into the cancer cells (64, 65). Inhibition of the glutaminase GLS1 and GLS2 either alone or in combination with other therapies enhanced the antitumor effects in preclinical studies (36, 37, 66–68). The tolerability and promising antitumor efficacy of the GLS1 small molecular inhibitor CB-839 combined with the vascular endothelial growth factor receptor (VEGFR)/MET inhibitor cabozantinib have been demonstrated in phase I/II clinical trials for patients with metastatic renal cell carcinoma (38–40). Overexpression of ASCT2 has been shown in various cancer types, including lung cancer (41), breast cancer (69), colorectal cancer (70), prostate cancer (71), and melanoma (72). Blockage of ASCT2 via monoclonal antibodies inhibits glutamine-dependent colorectal cancer cell growth *in vitro* and *in vivo* (42).

### Asparagine

Asparagine bioavailability significantly influences the metastatic potential of cancer cells. Asparagine serves as an essential amino acid for protein synthesis to adapt to the relatively low levels of extracellular glutamine in cancer cells (73). Suppression of the bioavailability of asparagine through dietary restriction or L-asparaginase, which catalyzes the hydrolysis of asparagine to aspartic acid and ammonia, suppresses breast cancer metastasis (43). L-asparaginase administration has been extensively used in treating acute lymphoblastic leukemia (ALL) because ALL cells cannot synthesize adequate levels of asparagine and highly depend on exogenous asparagine to maintain cell growth (44, 45). However, intolerable toxicities have been reported in patients treated with L-asparaginase in different clinical trials (74, 75), which may limit the clinical application of L-asparaginase in the treatment of solid tumors.

### Arginine

Arginine plays a crucial role in major physiological events, including cell proliferation, cell signaling, nitric oxide synthesis, and T-cell functions (76). Cancer cells are unexpectedly dependent on arginine for their growth, and depletion of arginine induced cancer cell death and tumor suppression in preclinical studies (77). Reduced expression of arginosuccinate synthase 1 (ASS1) has been observed in melanoma, glioma, lymphoma, and prostate cancer (78), and arginine deprivation therapy may generate antitumor efficacy in these cancer cells due to ASS1-involved arginine synthesis. A pegylated recombinant human arginase polyethylene glycol (PEG)-BCT-100 depleted systemic arginine. In advanced hepatocellular carcinoma (HCC) patients, PEG-BCT-100 demonstrated its safety and efficacy in a phase I trial (46). However, another arginine depletion therapy by administration with pegylated arginine deiminase ADI-PEG 20 as the second-line monotherapy in advanced HCC patients did not show a survival benefit in the phase III trial (47). In addition to cancer metabolism mediated by arginine, arginine is also involved in the immune escape of cancer cells (79) and immunomodulation of macrophages (80). Arginine deprivation therapy in combination with immunotherapy may be a rational modality for cancer treatment in the future.

## Nucleotide Metabolism

Adenosinergic molecules have been shown to activate immunosuppressive signals in tumor microenvironments. Inhibition of adenosinergic molecules is therefore a promising strategy for cancer treatment. Preclinical data showed that using the B7-DC/Fc fusion protein significantly improves the antitumor immune response in adenosine A_2A_ receptor (A2AR) knockout mice (81). The A2AR inhibitor SCH58261 combined with the anti-CD73 monoclonal antibody TY/23 generated synergistic antitumor effects and reduced cancer metastasis in a syngeneic mouse tumor model (48). A human monoclonal anti-CD73 antibody, MEDI9447, has been developed for anticancer treatment (49). The oral A2AR inhibitor CPI-444 in combination with immune checkpoint blockades, such as antibodies against programmed death 1 (PD-1) or cytotoxic T-lymphocyte-associated protein-4 (CTLA-4), generated synergistic antitumor effects and significant tumor regression and led to memory antitumor immune responses in preclinical studies (82). The safety and tolerability of CPI-444 alone or in combination with the anti-programmed death ligand 1 (PD-L1) antibody atezolizumab have been investigated in phase I clinical trials (50). The safety and efficacy of the humanized anti-CD73 monoclonal antibody CPI-006 administered alone or in combination with CPI-444 or pembrolizumab were studied in phase I clinical trials for advanced cancers (51).

## Lipid Metabolism

Endogenous fatty acid production is partially mediated by increased glycolytic metabolites in cancer cells (83). Frequent alterations of fatty acid synthase (FASN), a crucial metabolic multienzyme complex that is involved in the final process of fatty acid synthesis, have been revealed in various malignancies (84). FASN inhibition was proposed to induce antitumor activity by regulating apoptosis, cell membrane integrity, DNA replication (85), and Akt signaling (86, 87). The oral FASN inhibitor TVB-2640 combined with different cancer treatments has been assessed in clinical trials, including combined paclitaxel and trastuzumab for human growth factor receptor-2 (HER2)-positive advanced breast cancer (52), combined paclitaxel for patients with heavily pretreated breast cancer, and combined bevacizumab for high-grade astrocytoma (53). Acetyl-CoA carboxylase (ACC) catalyzes the rate-limiting step of fatty acid synthesis by carboxylation of acetyl-CoA to malonyl-CoA. Preclinical data showed that an inhibitor of ACC ND-646 administered alone or in combination with carboplatin exhibits antitumor efficacy in non-small cell lung cancer-bearing mice (54). Another liver-specific ACC inhibitor, ND-654, used alone or in combination with the multikinase inhibitor sorafenib, inhibited lipogenesis and cancer development in HCC-bearing rats (55). The antitumor efficacy of the lipid synthesis-targeting agents ND-646 and ND-654 are under investigation in clinical trials.

## TCA Cycle and Glucose Metabolism

The mutant *IDH2* inhibitor enasidenib has been approved by the U.S. Food Drug Administration (FDA) for the treatment of mutant *IDH2* recurrent or refractory acute myeloid leukemia (AML) (57). Furthermore, another mutant *IDH1* inhibitor, ivosidenib, was approved by the U.S. FDA for the treatment of mutant *IDH1* relapsed or refractory AML (58). Lipoate (lipoamide) is a cofactor that acts collaboratively with pyruvate dehydrogenase (PDH) and the α-ketoglutarate dehydrogenase (KGDH) complex in the TCA cycle. Elevated lipoate induces functional impairment of PDH through phosphorylation of PDH, which blocks the mitochondrial entrance of pyruvate to disrupt the TCA cycle (88). The small molecule lipoate analog CPI-613 not only inhibited the activities of PDH and KGDH but also exhibited antitumor effects in preclinical settings (59, 89). A phase I clinical trial showed that CPI-613 combined with chemotherapy maximizes the tolerated dose in patients with metastatic pancreatic cancer in a phase I clinical trial (60). Acidic tumor microenvironments caused by aerobic glycolysis are associated with metastasis, angiogenesis, and drug resistance (90). Targeting lactate through its transport, named monocarboxylate transporter (MCT), including MCT-1 and MCT-4 isoforms that are frequently expressed in cancer cells (91–94), is an anticancer strategy mediated through regulating the influx and efflux of lactate. The MCT-1 inhibitor AZD3965 combined with doxorubicin or rituximab showed synergistic antitumor efficacy in lymphoma *in vitro* (95). AZD3965 exhibited good tolerability and promising efficacy in preclinical studies and early-phase clinical trials of patients with advanced solid tumors (56).

### Applications of Cancer Metabolism as a Biomarker

The development of biomarkers for cancer metabolism is important for prognostication and assessment of metabolism-targeting treatment responses. However, the available metabolism-related markers are limited. Neomorphic *IDH* mutants are oncogenic drivers for generating the oncometabolite 2-hydroxyglutarate in AML and gliomas (21, 22). Recently, two mutant IDH inhibitors, enasidenib (AG-221) and ivosidenib (AG-120), have been approved for IDH-mutant refractory AML (57, 58). *IDH* mutation is therefore a biomarker for selecting IDH inhibitors. Another example is the *KEAP1* mutation as the biomarker for glumaminolysis inhibitors. Approximately 20% of *KRAS*-mutant non-small cell lung cancers carry loss-of-function mutations of the *KEAP1* gene (96). The major target of KEAP1 is Nrf2, which triggers antioxidative stress genes to endure oxidative stress (97). Enhanced Nrf2 activities further trigger the transcription of genes encoding antioxidants, drug pumping proteins, and other metabolic enzymes (98). In addition, increased glutamine addiction in cancer cells activates Nrf2. In a *KRAS*-mutant lung cancer mouse model, KEAP1 or Nrf2 mutations increased the sensitivity of glutaminase inhibitors (99). KEAP1 mutation may be considered a potential biomarker for anti-glutamine treatment. A further example is the application of the hexokinase isoform as a prognostic marker. The glycolytic enzyme hexokinase catalyzes glucose to glucose-6-phosphate, and high levels of hexokinase are expressed in cancer cells to accelerate glucose metabolism (100). Hexokinase isoform 2 has been identified as a prognostic biomarker in HCC, gastric cancer, and colorectal cancer (101).

In summary, deregulation of metabolism is important for cancer cells to adapt to rapid proliferation-induced environmental stress. Targeting cancer-specific metabolism is a promising therapeutic strategy, and future development of biomarkers for guiding this treatment is mandatory.

## Epithelial-Mesenchymal Plasticity in Cancer Progression

### Mechanism of EMT

In cancer metastasis, primary cancer cells acquire a mesenchymal phenotype to exhibit enhanced migration, invasion, and metastasis (102). In metastatic sites, tumor cells regain the epithelial phenotype for colonization to form secondary tumors via mesenchymal-epithelial transition (MET) (103). The expression levels of epithelial markers such as E-cadherin, epithelial cell adhesion molecule (EpCAM), cytokeratin, and occludin and mesenchymal markers including N-cadherin and vimentin are used to define the epithelial/mesenchymal status (104). We recently demonstrated that cancer cells in the intermediate status of EMT exhibit more aggressive properties to form collective cancer clusters to overcome stressful environments during metastasis (105).

EMT is regulated by multiple signaling networks via regulation at the levels of transcription, epigenetic regulation, translation, and post-translation (106). EMT transcription factors (EMT-TFs), including Snail, Twist1/Twist2, and the zinc finger E-box-binding homeobox (ZEB) families, are the major regulators of EMT (107). EMT-TFs act as transcriptional repressors to suppress the expression of epithelial genes; in addition, they act as activators to induce the transcription of mesenchymal and other metastasis-related genes (108). For example, Snail is a transcriptional repressor that induces EMT by suppressing E-cadherin. We showed that CREB-binding protein (CBP) acetylates Snail. Acetylation of Snail prevents the formation of a repressive complex and switches Snail from a repressor to an activator (109). Similarly, the bifunctional switch is also demonstrated in another important EMT transcriptional factor (EMT-TF), zinc finger E-box-binding homeobox 1 (ZEB1): it acts as a repressor of E-cadherin by binding to the E-boxes located at the *CDH*1 promoter (110). The interaction between the ZEB1 and Smad proteins and p300 also changes ZEB1 to an activator (111, 112).

In addition to transcriptional control, epigenetic regulation of epithelial and mesenchymal genes is crucial for providing plasticity and dynamic changes between epithelial and mesenchymal states. Snail recruits polycomb repressive complex 2 (PRC2) to repress *CDH1* expression by enriching the repressive mark H3K27me3 on the regulatory region (113). We demonstrated that Twist1 interacts with the polycomb repressive protein Bmi1 to act coordinately for EMT induction through suppression of *CDH1* and *P16INK4A* (114). MicroRNAs (miRNAs) are also involved in EMT by selectively suppressing the mRNAs of EMT-TFs by cleavage-mediated degradation or translational repression (115). The reciprocal regulation between ZEB1 and miR-200 family miRNAs plays an important role in maintaining epithelial-mesenchymal plasticity (116). The major external stimuli of EMT in cancer cells include TGF-β and hypoxic conditions. TGF-β is the best-known inducer that activates EMT-TFs to induce EMT (117, 118). We previously revealed that intratumoral hypoxia caused by the rapid proliferation of tumor cells activates EMT through the directional regulation of Twist1 by hypoxia inducible factor-1α (HIF-1α) (119).

### Functional Role of EMT in Metastasis and Treatment Resistance

Regarding the functional impacts of EMT on cancer cells, EMT induces stem-like properties (120) and enhances resistance to chemotherapy (3). Enriched cancer stem cells (CSCs) are shown in murine or human breast tumors with high expression of EMT-TFs (121). In colorectal cancers, we showed that Snail not only upregulates interleukin-8 (IL-8) to induce CSC formation (122) but also promotes the asymmetrical cell division-to-symmetrical cell division switch for expanding CSC pools (123). Both Snail and Twist1 conferred chemoresistance in a genetically engineered mouse model of pancreatic cancer (124). Twist1 and ZEB are commonly expressed in chemoresistant triple-negative breast cancers (125). Snail contributes to cisplatin resistance by upregulating the DNA repair protein excision repair cross complementation group 1 in head and neck cancer (126). Various EMT signatures have been shown to predict resistance to epidermal growth factor receptor (EGFR) or phosphatidylinositol 3-kinase (PI3K) inhibitors in clinical samples and cell lines derived from non-small-cell lung cancer patients (127). In summary, dynamic changes between epithelial and mesenchymal states are crucial for metastasis and the malignant characteristics of cancer cells, such as cancer stemness and therapeutic resistance.

### Feasibility of EMT as a Therapeutic Target and Biomarker

EMT is an attractive target for antimetastatic therapy owing to the significant contribution of EMT to metastasis (128). However, inhibiting EMT may simultaneously promote MET, which is a crucial step for metastatic colonization. Ideally, specific killing of metastatic mesenchymal-type cancer cells will be effective; however, extensive drug resistance has been noted in these cells. In addition, distinct mechanisms for inducing EMT hinder the development of anti-EMT therapy. Theoretically, anti-EMT therapies include precise targeting of mesenchymal-type cancer cells and reversing EMT/transdifferentiation of EMT cancer cells into innocuous cells (128). Direct targeting of EMT-undergoing cancer cells is relatively difficult because drug resistance frequently exists in EMT cancer cells, and EMT-TFs are mostly undruggable. An alternative approach to intercept EMT is to target the downstream signals of EMT. For example, miR-200 family microRNAs downregulate the metastasis-inhibiting secretory protein tubulointerstitial nephritis antigen-like 1 (Tinagl1) during MET. Treatment with recombinant Tinagl1 suppressed triple-negative breast cancer progression and metastasis (129).

EMT-induced mesenchymal markers on circulating tumor cells (CTCs) are also potential biomarkers for cancer metastasis (130). For example, cell-surface vimentin (CSV) is detected on CTCs from the blood of patients with metastatic colon cancer. CSV expression is significantly higher in metastatic tumors than in primary tumors, implying that CSV expression is correlated with colon cancer metastasis and could serve as a metastatic biomarker (131). CSV-positive CTCs also serve as a diagnostic and prognostic biomarker in pancreatic cancer (132). Moreover, the other EMT-induced CTC marker, plastin3 (PLS3), which acts as an actin-bundling protein known to inhibit cofilin-mediated depolymerization of actin fibers, is a prognostic biomarker in colorectal cancer (133) and breast cancer (134). Recently, EMT has also been associated with the immunosuppressive tumor microenvironment (135) and thus has become a potential biomarker to predict the responses to PD-1/PD-L1 blockade immunotherapy (136).

Together, anti-EMT therapies should be rationally combined with other modalities of cancer treatments to maximize antitumor efficacy. Identification of more druggable targets of EMT as cancer therapeutics will be mandatory for the development of anti-EMT treatment.

## Interplay Between Cancer Metabolism and Epithelial-Mesenchymal Plasticity

### Evidence of Their Mutual Influences

**Figure 1:**
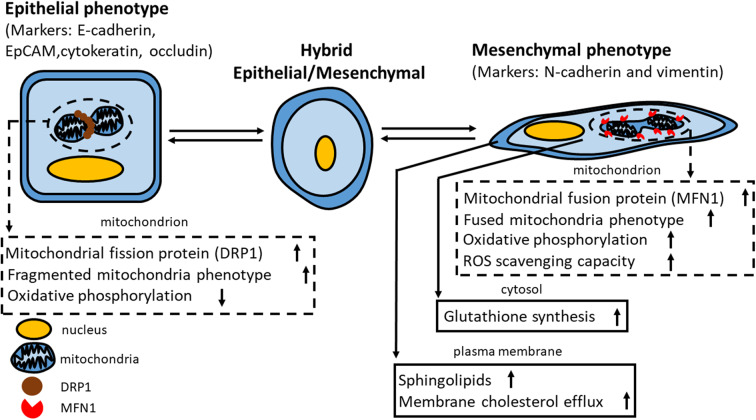
Correlation between cancer metabolism and epithelial-mesenchymal plasticity. The schema presents the differential characteristics of metabolism in cancer cells in the epithelial, mesenchymal, and hybrid states. MFN1, mitofusin-1; ROS, reactive oxygen species; DRP1, dynamin-related protein-1. Please note that the size of mitochondria is disproportionately magnified for presenting the molecular events in epithelial/mesenchymal states.

Accumulating evidence indicates the distinct energy requirements in different steps of metastasis. A favorable reprogramming of metabolism has been noted to provide a survival advantage for metastatic cancer cells such as CTCs by prioritizing energy production (102). Adaptation of metabolism was shown to modulate cancer cell motility through mitochondrial regulation (137) as well as detachment of cancer cells from the extracellular matrix (138) and invasion (139). The major EMT inducer transforming growth factor-β (TGF-β) also affects various cancer metabolic processes, such as glycolysis, mitochondrial respiration, and lipid metabolism (117, 118). The major metabolic changes of the different epithelial/mesenchymal states can be summarized as several categories, including mitochondrial dynamics, lipid metabolism, and the influence of cell-matrix interactions on energy production. Figure 1 summarizes the major metabolic events in different epithelial-mesenchymal states of cancer cells.

The maintenance of mitochondrial morphology is balanced by mitochondrial fission and fusion (140–142). Fragmented mitochondrial formation is mediated by either enhanced mitochondrial fission or repressed mitochondrial fusion through fission or mitofusin-related proteins, respectively. Epithelial or mesenchymal cells display distinct mitochondrial morphologies that regulate their mitochondrial function for cellular proliferation. In epithelial-type cancer cells, the mitochondrial fission protein dynamin-related protein 1 (DRP1) mediates fragmented mitochondria (141, 143) and impaired oxidative phosphorylation (144, 145). Recently, Wu et al. demonstrated that Snail and TGF-β direct mesenchymal cells to display fused/tubulated mitochondria via activation of the mitochondrial fusion protein mitofusin-1 (MFN1), which enhances glutathione synthesis and the ROS scavenging capacity in mammary stem cells (137). Together, fused mitochondria are associated with increased oxidative phosphorylation and the TCA cycle (146, 147), and a reversal of the EMT phenotype is correlated with decreased fused mitochondria with impaired mitochondrial function (137).

TGF-β1 induces EMT in cancer cells through the induction of EMT-TFs such as Snail (117, 118). In addition to inducing EMT-TFs, TGF-β1 directs the metabolic switch from glycolysis to oxidative phosphorylation through suppression of pyruvate dehydrogenase kinase 4, which acts as a checkpoint of TCA cycle entry by repressing the activity of pyruvate dehydrogenase (148). In addition, TGF-β1-induced EMT and metastasis suppress lipogenesis and enhance oxidative phosphorylation in lung cancer (149). Cancer cell migration, invasion, and metastasis enhance oxidative phosphorylation and mitochondrial biogenesis mediated by upregulation of the transcriptional coactivator peroxisome proliferator-activated receptor gamma coactivator 1 alpha (PGC-1α) in breast cancer (150). However, contradictory studies have shown that Snail facilitates glucose uptake, macromolecule biosynthesis, and respiration inhibition by repressing fructose-1,6-bisphophatase 1 in basal-like breast cancer (151). In addition, Snail was shown to promote glycolytic metabolism by inhibiting phosphofructokinase (152) or cytochrome C oxidase (153) in breast cancer. Therefore, the role of EMT in regulating cancer metabolism is still controversial.

Metastatic cancer cells acquire their motile and invasive capacity to detach from primary tumors, enter into and leave the bloodstream, and colonize to form metastatic tumors. Metabolic alterations of lipids have been shown to be involved in these processes. Alterations of structural components of the cell membrane, including lipid rafts, cholesterol, and sphingolipids, together with regulators of cellular motility/invasiveness, such as CD44, extracellular matrix (ECM) as a degraded non-cellular structure, and invadopodia formation enhance cancer cell motility (154). High levels of cholesterol in membranes impede cellular fluidity and subsequently decrease metastasis by limiting cell motility during EMT and intra/extravasation (155). Cancer cells upregulate the ATP-binding cassette transporter ABCA1 to increase cholesterol efflux, and overexpression of ABCA1 in human cancers increases metastasis (155). Sphingolipids enhance EMT (156) and the motile phenotype (157, 158) by modulating sphingosine-1-phosphate receptor-dependent or receptor-independent signaling pathways in cancer cells. In addition, various enzymes of lipid metabolism are involved in EMT of cancer cells (159). For instance, the lipogenic enzyme ATP-citrate lyase (ACLY) has been shown to interact with the low molecular weight isoform of cyclin E to promote the transformation, migration and invasion of breast cancer cells (160).

The absence of cell-matrix interactions induces a type of programmed cell death, anoikis (161), which occurs during the detachment of non-hematopoietic cells from the ECM due to insufficient glucose uptake-induced shortage of ATP (162). In contrast, CTCs rewire their metabolism not only to prevent anoikis but also to enhance the acquisition of anchorage independence during metastatic dissemination (163, 164). CTCs diminish the levels of ROS caused by ECM detachment to evade anoikis (162, 165). In lung cancer cells, the detachment process suppresses both cell growth pathways and carboxylation of cytosolic α-ketoglutarate and then induces citrate into mitochondria to further enhance NADPH production to relieve oxidative stress (166). Elevated ROS levels are also found in melanoma CTCs compared to their primary tumors. To withstand oxidative stress, metastatic melanoma cells undergo reversible metabolic alterations to enhance NADPH-generating enzymes through the folate pathway (167). Together, alterations of cell-matrix interactions increase the oxidative stress of metastatic cancer cells. Cancer cells undergo metabolic reprogramming to overcome oxidative stress to sustain survival.

### Potential Therapies for Intercepting EMT Through Metabolic Targeting

EMT is highly associated with cancer metastasis through alterations of multiple crucial events, including metabolic rewiring (168). For amino acid metabolism, cancer cells mainly utilize glutamine to synthesize nucleotides and nonessential amino acids as well as to provide substrates for the TCA cycle (34). Glutamine metabolism has been reported to regulate EMT. Inhibition of glutaminolysis, the deamination process of glutamine into glutamate, by targeting glutaminase GLS1 alleviates cancer metastasis by suppressing Snail in lung cancer (169). The mitochondrial isoform of glutaminase GLS2 inhibits migration, invasion, and metastasis through repression of Snail in HCC (170). These findings suggest that targeting glutaminase in cancer cells not only blocks glutamine addiction but also suppresses EMT.

Cancer cells increase lipogenesis and lipolysis to rewire lipid metabolism. The fatty acid synthetic enzyme FASN induces EMT by enhancing TGF-β expression in non-small cell lung cancer (171). In addition, EMT promotes FASN expression. FASN and EMT are reciprocally and coordinately upregulated in non-small cell lung cancer (171). The FASN inhibitor TVB-2640 may inhibit tumor growth and metastasis, and the efficacy is under evaluation in clinical trials. In addition, peroxisome proliferator-activated receptor (PPAR) family members, regulators of fatty acid synthesis and oxidation, are involved in lipid synthesis or degradation as well as EMT inhibition (172). Pharmacological and genetic inhibition of PPARβ/δ increased metastasis through EMT induction in melanoma (172). Moreover, PPARγ knockout induced both EMT and stemness in prostate cancer (173). These studies indicate that targeting PPARβ/δ or γ may have potential clinical applications for the treatment of cancer patients by reducing metastasis. In addition to the FASN and PPAR members, a membranous lipid with a sphingosine backbone, sphingolipid, as described earlier, is also involved in EMT induction by regulating sphingosine kinase 1 (SPHK1), which converts sphingosine into sphingosine 1-phosphate (S1P) (174, 175) in cancers. SPHK1 has been reported to induce EMT through autophagic degradation of E-cadherin (176) or activation of focal adhesion kinase (177) in HCC or colorectal cancer, respectively. S1P has a role in EMT induction via the matrix metalloproteinase-7 (MMP-7)/TGF-β autocrine loop in HCC (178). Therefore, SPHK1 and S1P may serve as promising therapeutic targets to alleviate EMT-mediated metastasis by disrupting sphingolipid metabolism in cancers.

In glucose metabolism, cancer cells increase glucose uptake and glycolysis flux, mitochondrial dysfunction, or the acidic tumor microenvironment to promote progression. For example, MMP-2 expression is increased by the major glucose transporter GLUT1 to further enhance EMT and invasion in cancer cells (179, 180). Consequently, cancer-specific GLUT1 targeting may reduce metastasis in cancers. In summary, EMT-mediated cancer metastasis may be attenuated by targeting cancer metabolites, including amino acids, lipids, and glucose, as a potential alternative anti-cancer modality.

## Conclusions

Metabolic reprogramming and epithelial-mesenchymal plasticity are the major adaptive strategies of cancer cells to endure rapid proliferation and metastasis-related environmental stress. Understanding the dynamic changes of the epithelial and metabolic states during the metastatic process is essential for developing optimal strategies to target disseminated cancers. Although a growing number of drugs have been developed to target cancer-specific metabolism, there are still issues that must be addressed before wide application of this treatment. Discovery of biomarkers for guiding antimetabolic treatment and elucidation of the interplay between metabolism and EMT to prevent metabolic change-induced adverse events will be important for the treatment of highly dynamic metastatic cancers.

## Author Contributions

N-YS and M-HY wrote the manuscript and approved the final version.

## Conflict of Interest

The authors declare that the research was conducted in the absence of any commercial or financial relationships that could be construed as a potential conflict of interest.
